# Potency of *Artocarpus Heterophyllus* Leaves Extract as an Antiviral against SARS-CoV-2

**DOI:** 10.24546/0100503458

**Published:** 2026-04-02

**Authors:** ADITA AYU PERMANASARI, LIDYA TUMEWU, ALDISE MARETA NASTRI, MARIA ISTIQOMAH MARINI, ACHMAD FUAD HAFID, TUTIK SRI WAHYUNI, MITSUHIRO NISHIMURA, MARIA INGE LUSIDA, ATY WIDYAWARUYANTI, YASUKO MORI

**Affiliations:** 1 Center of Natural Product Medicine Research and Development, Institute of Tropical Disease, Universitas Airlangga, Surabaya, Indonesia; 2 Division of Clinical Virology, Center for Infectious Diseases, Kobe University Graduate School of Medicine, Kobe, Japan; 3 Department of Pharmaceutical Sciences, Faculty of Pharmacy, Universitas Airlangga, Surabaya, Indonesia; 4 Department of Medical Microbiology, Faculty of Medicine, Universitas Airlangga, Surabaya, Indonesia

**Keywords:** *Artocarpus heterophyllus*, Antiviral, COVID-19, SARS-CoV-2, Infectious disease, Medicine

## Abstract

Natural products, particularly those derived from plants, represent a valuable source of bioactive compounds with potential antiviral activity. In this study, we investigated the antiviral activity of Indonesian plants which belong to *Artocarpus* genus against SARS-CoV-2 using bioassay-guided separation. Plant materials from the *Artocarpus* genus were successively extracted using hexane, dichloromethane, and methanol, and the antiviral activities were tested against early SARS-CoV-2 variants. Viral inhibition and cytotoxicity were assessed using RT-qPCR, plaque assays, and MTT assays. Among 54 extracts obtained from the leaves and stem bark of 10 species of Artocarpus, four dichloromethane leaf extracts inhibited viral RNA replication and cytopathic effect at a concentration of 10 μg/mL, and did not show cytotoxic effects at a concentration of 100 μg/mL. The most potent extract was dichloromethane leaf extract of *Artocarpus heterophyllus* (E16) with the 50% inhibitory concentration (IC_50_) value of 2.46 μg/mL. Separation by vacuum liquid chromatography and open column chromatography yielded E16F4.3 and E16F4.4 which possess potential activity against SARS-CoV-2 with IC_50_ values of 0.43 μg/mL and 0.28 μg/mL. These subfractions showed less cytotoxicity, with 50% cytotoxic concentration (CC_50_) values >100 μg/mL. The selective index (SI; CC_50_/IC_50_) of E16F4.3 and E16F4.4 were >232.6 and >357.1, respectively. These results indicate that the leaf extract of *Artocarpus heterophyllus* contains promising drug candidates against SARS-CoV-2.

## INTRODUCTION

Severe acute respiratory syndrome coronavirus 2 (SARS-CoV-2) is a novel coronavirus that was first reported in Wuhan, Hubei province, China, in December 2019 (1, 2), and caused coronavirus disease 2019 (COVID-19). The first identified variant carried a single-point mutation in the spike protein (D614G) (3), and its descendants such as B.1.468 and B.1.470 variants (4) were detected in Indonesia during 2019–2020. Following the widespread transmission of Omicron variants and their derivatives, most therapeutic monoclonal antibodies lost their efficacy (5, 6), while resistant mutants against small-molecule drugs such as Nirmatrelvir and Remdesivir are also reported (7, 8). Some studies have reported the detection of drug resistance mutations, especially in individuals who received Nirmatrelvir or Remdesivir and immunosuppressive therapy (9). Because SARS-CoV-2 is prone to mutation, antiviral drugs need to account for the potential emergence of resistant mutants, and thus, ongoing exploration of new therapeutics is required.

SARS-CoV-2 has a single-stranded, positive-sense RNA genome, and the virion consists of four structural proteins: spike (S), envelope (E), membrane glycoprotein (M), and nucleocapsid phosphoprotein (N) (10, 11). Remdesivir targets the replication machinery of SARS-CoV-2, i.e., RNA-dependent RNA polymerase (RdRp), and was the first drug approved by the Food and Drug Administration (FDA) for the treatment of COVID-19. Ritonavir-boosted Nirmatrelvir (Paxlovid), targeting the 3C-like protease (3CLpro), and Molnupiravir targeting the RdRp have also received Emergency Use Authorizations from the FDA for the treatment of COVID-19 (10, 12, 13). Inhibitors of cysteine papain-like protease, PLpro (14) and M protein (15, 16) are also considered promising drug targets, although they are still under development. By exploring a broad range of sources for potential drug candidates, it is possible to identify novel agents that inhibit SARS-CoV-2 replication.

Previous studies reported that the genus *Artocarpus* has potential pharmacological effects due to its metabolites. The *Artocarpus* genus is a member of the *Moraceae* family, with around 50 species and a wide distribution in ASEAN countries. In Indonesia, *Artocarpus* has been found in 5 large islands with a total of 30 species, including in Java, Kalimantan, Sumatra, Sulawesi, and Maluku, with 23 species in Borneo (Kalimantan), which is estimated to be the center of the world’s *Artocarpus* diversity (17, 18). The *Artocarpus* genus has been reported as a potential source of bioactive compounds. An extract from *A. heterophyllus* has shown antiviral activity against Hepatitis C virus (HCV) with the IC_50_ value of 1.5 μg/mL (19, 20). C-geranylated flavonoid, Artocarones, isolated from *A. communis* leaves, was reported to exhibit inhibitory activity against Herpes Simplex Virus type 1 (HSV-1) (21). Oxyresveratol, isolated from the heartwood of *A. lakoocha*, showed moderate activity against HSV-1, Herpes Simplex Virus type 2 (HSV-2), and Human Immunodeficiency Virus (HIV), with an IC_50_ of 28.2 μM (22). Furthermore, Artocarpin, isolated from several *Artocarpus* species (*A. Heterophyllus, A. champeden, A. anisophyllus, A. altilis*, and *A. chama*) showed potential as inhibitors against Influenza virus Neuraminidase (NA) (23, 24).

In this study, 54 extracts from 10 species of the *Artocarpus* genus were analyzed for antiviral activity against SARS-CoV-2, and the results showed that two subfractions of the *A. heterophyllus* leaf extract exhibited potent antiviral activity against SARS-CoV-2, suggesting their potential as a source of novel antiviral agents.

## MATERIALS AND METHODS

### Extraction of Artocarpus plant materials

The ten plant species belonging to the *Artocarpus* genus were collected from Purwodadi Botanical Garden, East Java, Indonesia, in February 2023, from locations IV (B, C, D) and V (G), yielding 1–3 kg of fresh leaves and stems. The names of the species are *A. altilis, A. elasticus, A. fratessi, A. heterophyllus, A. integer, A. rigidus, A. sericicarpus, A. sp, A. tamaran, and A. teysmanii*. The leaves and stems/stembarks were dried at room temperature and then ground into a fine powder before being extracted using successive maceration, a method that employs solvents of increasing polarity. Three solvents were used in this study: hexane, dichloromethane (DCM), and methanol. A total of 25 g of dried leaves or stems/stembarks was sonicated for 2 minutes, three times, with 1 L of hexane to produce a hexane extract. This extraction method was repeated three times (400 mL, 300 mL, 300 mL). The resulting residue was subsequently extracted with dichloromethane to obtain the DCM extract and the remaining residue was re-extracted with methanol to produce the methanol extract. The extracts were concentrated using a rotary evaporator at 40°C. In total, 54 extracts were obtained and the dried extracts were stored at 4°C until further use.

### Cell culture and virus propagation

The cells used in this study were Vero cells and Vero E6/TMPRSS2 cells (25). Vero cells and Vero E6/TMPRSS2 cells were propagated in the Dubelco’s Modified Eagle Medium (DMEM GIBCO) supplemented with 10% Fetal Bovine Serum (FBS; Sigma Aldrich) and 1% of penicillin-streptomycin solution (GIBCO). Vero E6/TMPRSS2 cells were cultured in the same medium with the addition of 1 mg/mL G418 (Nacalai Tesque) to maintain TMPRSS2 expression. The cells were maintained in a 10-cm dish at 37°C in an incubator with 5% CO_2_. Cells were passaged when they reached >95% confluency.

SARS-CoV-2 strains used in this study included the East Java variant (B1.470; GISAID accession ID EPI_ISL_529965), obtained from the Institute of Tropical Disease, Airlangga University, and the Biken-2 variant (B-2; DDBJ accession number ID LC644163), which carries only the D614G mutation in the spike gene. Briefly, Vero cells (5 × 10. cells per well) were seeded into 6-well plates 24 hours before infection. The virus stock, B1.470 stock was diluted in 3 dilutions: 100-fold, 1000-fold, and 10,000-fold, in DMEM without FBS. Trypsin-TPCK was included at a final concentration of 2.5 μg/mL to support the growth of virus. The solution was then inoculated onto 6-well plate and incubated for 6 days. Supernatant containing the virus was collected, and debris cells were removed by centrifugation at 3000 × *g*, 10 min, 4°C at 6 days post-infection (dpi), and the virus solution was mixed with 0.2% BSA in TGS buffer at a 1:2 ratio, then kept at −80°C deep freezer until use. Furthermore, the Biken-2 variant was propagated in Vero E6/TMPRSS2 cells using DMEM containing 2% FBS as previously described (26, 27). All experiments using SARS-CoV-2 were conducted in Biosafety Level 3 (BSL-3) laboratories at the Institute of Tropical Disease, Airlangga University, and Kobe University Graduate School of Medicine.

### Determination of viral titer by TCID_50_ and plaque assay

After virus propagation, we determined the virus concentration using two methods: the tissue culture infectious doses 50 percent (TCID_50_) and Plaque Forming Unit (PFU/mL). For the TCID_50,_ virus was diluted in the medium at 10-fold dilutions (10^−1^ to 10^−9^) and inoculated into 5 replicate wells. The TCID_50_ concentration was counted and determined using the Reed and Muench method (28). In brief, CPE-positive and -negative wells were recorded across the dilution range, and the 50% endpoint was calculated by interpolation between the dilution that gave a 50% infection rate. For the plaque assay, cells were prepared in a 12-well plate at a density of 2 × 10^5^ cells/well and incubated overnight (20–24 hours) at 37°C in the incubator with 5% CO_2_. Culture medium was discarded from each well, then the cells were washed with 1 mL DMEM. The virus was serially diluted from 10^−1^ to 10^−8^, and 200 μL of the diluted virus was inoculated into the cells. After incubation for 1 hour at 37°C in 5% CO_2_ with rocking every 5 minutes, the medium was removed. Plates were washed twice with Phosphate-Buffered Saline (PBS), and 2 mL of DMEM supplemented with 0.7% methyl cellulose was added to each well, and then incubated for 4–6 days at 37°C in incubator with 5% CO_2_. Cells were fixed with 80% methanol for 60 minutes, then 1% crystal violet solution (500 μL/well) was added. The plaques were calculated as the number of plaques divided by the dilution factor and the plated volume, and expressed as PFU/mL.

### Evaluation of Anti-SARS-CoV-2 activity

Vero cells were seeded at a density of 2 × 10^4^ cells per well 24 hours prior to virus infection in a 48-well plate. The cells were washed with 200 μL of DMEM without FBS. Stock solutions of each extract were prepared at a concentration of 100,000 μg/mL in DMSO, and diluted to 200, 100, 20, 10, 0.2, and 0.02 μg/mL for extracts and fractions, and 200, 20, 2, 0.2 μg/mL for subfractions using DMEM without FBS. As a positive control, we prepared Remdesivir (REMDAC, Cadila Healthcare-Limited, India) at 20 μM. The virus (East Java variant, B1.470) solution was prepared at a concentration of 200 TCID_50_ in DMEM containing 5 μg/mL trypsin TPCK. A mixture of sample and virus was prepared by combining 400 μL of virus solution and 400 μL of each sample solution, followed by incubation for 1 hour at 37°C. Then, 200 μL of the virus-sample mixture was inoculated into cells in duplicate wells. The cytopathic effect (CPE) was evaluated at 6 dpi and scored as follows: Score 1: 0–25% CPE; Score 2: 26–50% CPE; Score 3: 51–75% CPE; Score 4: 76–99% CPE; and Score 5: 100% CPE.

At 6 dpi, the supernatant from each well was subjected to RNA extraction using the QIAamp viral mini kit (Qiagen, Valencia, California) according to the manufacturer’s protocol, and RNA was eluted with 60 μL of nuclease-free water. Reverse transcriptase real-time PCR for the Orf1ab and E genes was performed using the Standard M nCoV Real-Time Detection kit (SD Biosensor, Republic of Korea). The reaction mixture was prepared in triplicate according to the manufacturer’s protocol, using 5 μl RNA eluate. RT-PCR was performed on an Applied Biosystems 7500 Fast system (Applied Biosystems, Massachusetts, USA). The reaction conditions were as follows: step 1, Reverse transcription was carried out at 50°C for 15 min, followed by initial denaturation at 95°C for 3 min. Amplification was performed for 45 cycles at 95°C for 5 sec and 60°C for 40 sec. RNA expression levels of Orf1ab and E genes were estimated based on the cycle threshold (Ct) values as 2^(−Ct)^. Infection rate was calculated by dividing RNA expression level of each sample by that of the negative control (0.1% DMSO). For dose-dependent analysis, the IC_50_ was defined as the concentration at which the RNA expression level was 50% of the negative control. The IC_50_ value was estimated by log-linear interpolation between the two points on either side of the 50% response.

### Cell viability assay using MTT method

Vero cells were seeded at a density of 1 × 10^4^ cells per well in a 96-well plate and incubated for 24 hours. Sample solution (100,000 μg/mL in 100% DMSO) was diluted to 100, 50, 10, 1, 0.1, and 0.01 μg/mL with DMEM with 10% FBS, and added to the wells. The plate was incubated at 37°C in 5% CO_2_ for 3 days. DMEM with 10% FBS and 0.1% DMSO was used as the negative control. After incubation, the medium was discarded, and 150 μL of 10% 3-(4,5-dimethylthiazol-2-yl)-2,5-diphenyltetrazolium bromide (MTT) reagent was added to each well. To maximize the MTT enzyme reaction, the plate was incubated for 4 hours, followed by the addition of 100 μL of 100% DMSO to dissolve the formazan crystals. Cell viability was determined by measuring absorbance at 560 nm using a microplate reader (Thermo Scientific™ Multiskan SkyHigh), and background absorbance at 750 nm was subtracted. The percentage of cell viability was calculated by dividing the absorbance values of treated wells by those of the negative control (0.1% DMSO). The 50% cytotoxicity concentration (CC_50_) was estimated as the highest concentration at which the cell viability exceeded 50%.

### Separation of active extract by vacuum liquid chromatography and column chromatography

The selected active extract, E16, was separated by vacuum liquid chromatography and evaluated for its activity to obtain active fractions as anti-SARS-CoV-2 activity. A number of 27 combinations of mobile phases (n-hexane, dichloromethane, ethyl acetate, and methanol) were used. In brief, 2.5 g of the active extract was separated on 25 g of Silika gel 60 using 30 mL of the mixed solvent for each combination. Fractions were collected by vacuum, resulting in 27 fractions. These fractions were analyzed by Thin-Layer Chromatography (TLC), and the fractions with similar profiles were combined, yielding 6 fractions, designated E16F1–E16F6. An antiviral assay against SARS-CoV-2 was conducted on these fractions to identify the active ones. The most active fraction, E16F4, was further separated using column chromatography. In brief, 200 mg of E16F4 was fractionated using three combinations of solvents: i.e., acetonitrile (ACN):Water 4:1 v/v; ACN:Water 9:1 v/v; and 100% ACN. These separations resulted in 9 subfractions after TLC profiling. The subfractions were stored at 4°C until use.

### Plaque reduction assay

For the antiviral plaque reduction assay, 2 × 10^5^ of VeroE6/TMPRSS2 cells were seeded in each well of a 12 well plate 24 hours prior to infection. Sample stock solutions (20,000 μg/mL in 100% DMSO) were diluted to 2 μg/mL with DMEM without FBS. Virus solution (Biken-2 variant; B-2) at 4 × 10^3^ PFU/mL was mixed with each diluted sample at 1:1 ratio, and incubated at 37°C for 1 hour. The medium in the cell plate was removed, and cells were washed with DMEM without FBS. Then, the virus-sample mixture was added to two replicate wells containing cell monolayers at a final virus titer of 200 PFU/well. The plate was incubated for 1 hour at 37°C, with gentle shaking every 5 minutes. After incubation, the mixture was removed, and cells were washed twice with PBS. The methylcellulose 0.7% in DMEM with 2% FBS containing each diluted sample was added and incubated for 3–6 days. Cells and virus were fixed and inactivated using 80% methanol for 1 hour. The plaque was stained by adding 500 μL of 1% Crystal violet in 50% methanol, and the visualized plaques were counted.

## RESULTS

### Screening of Artocarpus extracts reveals four candidates with antiviral activity against SARS-Cov-2

Among 10 species of *Artocarpus* genus, a total of 54 extracts (E1–E54) were evaluated against SARS-CoV-2. The antiviral activity against an early SARS-CoV-2 variant (B.1.470 isolated in Indonesia during 2019–2020) was assessed at a concentration of 10 μg/mL at 6 dpi through two methods: Reverse transcription quantitative-PCR (RT-qPCR) to measure viral genomic replication, reported as cycle threshold (Ct) values for the E and Orf1ab genes in the supernatant, and Cytopathic effect (CPE) scoring to determine the infectivity in the cells (Table I). The results showed that only four active extracts from leaf DCM extracts inhibited SARS-CoV-2; i.e., *Artocarpus fratessi* leaves DCM extract (E13); *Artocarpus heterophyllus* leaves DCM extract (E16); *Artocarpus integer* leaves DCM extract (E22); and *Artocarpus sp*. leaves DCM extract (E40). These four extracts showed Ct values >25 and the CPE score 1, indicating suppressed genome replication and complete viral inhibition (Table I). To determine whether the inhibition was affected by cytotoxicity, cell viability in the presence of each extract at 100 μg/mL was analyzed using the MTT assay (Table I). Six extracts showed cell viability <50% (E4, E7, E25, E28, E46, and E52). Among the four active antiviral extracts, E13, E16, and E22 showed no cytotoxicity (viability >80%), while E40 exhibited moderate toxicity (viability = 51.9%).

### Antiviral activity and cytotoxicity of E16 subfraction

**Figure 1:**
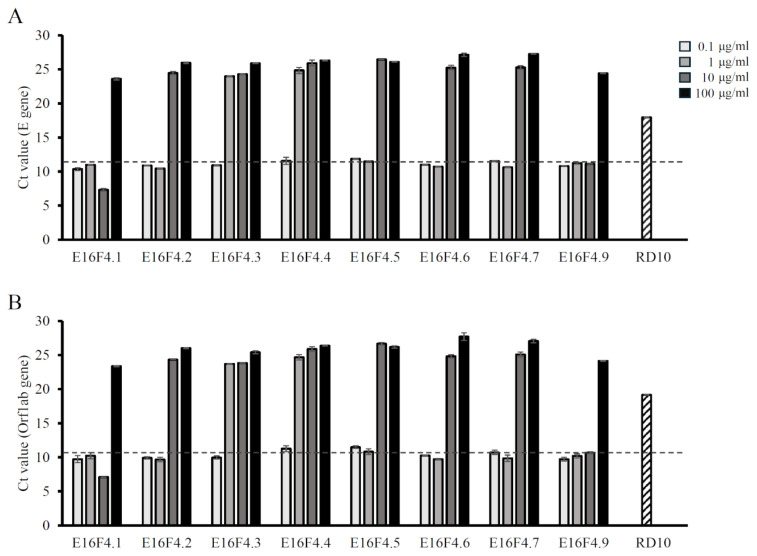
Dose-response evaluation of subfractions from E16F4 for antiviral activity against SARS-CoV-2 B.1.470. (A, B) The mean Ct values of E gene (A) and Orf1ab gene (B) ± standard deviation (SD) from two wells of a single experiment were plotted. A reference Ct value of the negative control (0.1% DMSO) is indicated by a horizontal line. Ct values above the control line indicate a reduction in viral RNA. As a positive control, value of 10 μM Remdesivir (RD10; approximately 6.0 μg/mL, assuming a molecular weight of 602.6 Da) is also shown, for reference.

One of the candidate extracts, E16, was subjected to further separation by vacuum liquid chromatography. The first round of separation by column chromatography yielded a bioactive fraction, E16F4, and the second round of isolation resulted in nine subfractions (E16F4.1–E16F4.9). Dose-dependent antiviral activities of these subfractions are shown in Figure 1, except for E16F4.8, for which data were insufficient. The antiviral effect of subfractions E16F4.1 to E16F4.9 and a positive control of Remdesivir (RD10; 10 μM, approximately 6.0 μg/mL) was assessed by RT-qPCR, targeting two SARS-CoV-2 genes: the envelope (E) gene and the ORF1ab gene. Treatment with the subfractions resulted in increasing Ct values for the E gene compared to the negative control 0.1% DMSO (horizontal line), indicating a reduction in viral RNA. Subfractions E16F4.3 and E16F4.4 demonstrated potent activity, with Ct values of approximately 20–25 at 1 μg/mL, which are comparable Ct value achieved by the positive control Remdesivir (RD10). A similar pattern of inhibition was observed for the ORF1ab gene (Figure 1).

**Figure 2:**
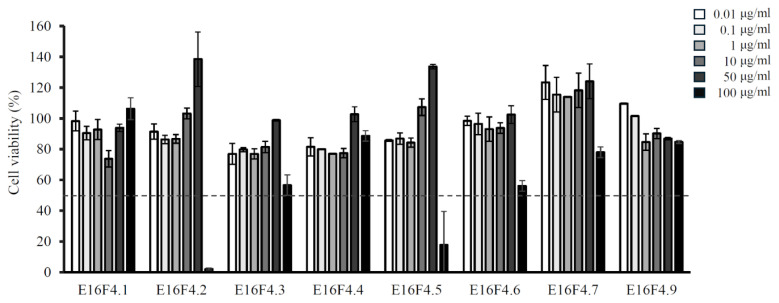
Cytotoxicity profiles of the subfractions on Vero cells evaluated by the MTT assay. Cell viability was calculated as a percentage relative to the negative control (0.1% DMSO). The 50% viability threshold is indicated by a horizontal line. Each data point represents the mean value ± standard deviation (SD) from two technical replicates in a single experiment.

Two subfractions, E16F4.3 and E16F4.4, exhibited potent antiviral activity, with the estimated IC_50_ values of 0.43 μg/mL and 0.28 μg/mL, respectively (Table II). Cell viability was evaluated using the MTT assay (Figure 2). All subfractions showed minimal cytotoxicity, with CC_50_ values exceeding 50 μg/mL for E16F4.2 and E16F4.5, or over 100 μg/mL for the remaining fractions (Table II). The selectivity index (SI) was highest for E16F4.3 (>232.6) and E16F4.4 (>357.1). These data indicated that E16F4.3 and E16F4.4 contain anti-SARS-CoV-2 compounds with low toxicity. Moderately active subfractions included E16F4.5, E16F4.6, E16F4.7, and E16F4.2 (IC_50_ = 2.96–5.52 μg/mL), whereas E16F4.9 and E16F4.1 exhibited weak activity, with IC_50_ values of 37.93 μg/mL and 93.37 μg/mL, respectively.

The plaque reduction assay confirmed the antiviral activity of these subfractions (Table III). Subfraction E16F.4.5 achieved complete viral inhibition (100%) at 1 μg/mL, whereas E16F.4.4 showed a comparable effect (97.6%). E16F4.2, E16F4.3, E16F4.6, and E16F4.7 exhibited substantial antiviral activity, with inhibition rates ranging from 89.0% to 92.4% under the same conditions. E16F4.1 and E16F4.9 demonstrated moderate activity, at 75.3% and 69.5%, respectively. Although the antiviral activity trend observed in the plaque reduction assay did not fully align with the RT-qPCR results, both assays consistently identified that E16F4.3 and E16F4.4 as promising candidates for further investigation.

## DISCUSSION

Nature has provided numerous herbs and plant extracts that have been used to treat a wide range of diseases. The global use of herbal and other natural products continues to rise, driven by consumers’ preference for natural therapies and concerns regarding the adverse side effects of modern medicines. Plants are recognized as rich sources of therapeutic agents and have served as the basis for the development of several synthetic drugs. Despite significant advances in organic synthesis, approximately 75% of prescribed drugs worldwide are derived from plant sources (29–31).

In this study, we identified antiviral activity against SARS-CoV-2 B.1.470 in species of the genus *Artocarpus*. This provides the first *in vitro* experimental evidence of anti-SARS-CoV-2 activity from *Artocarpus* extracts, although several prior *in silico* studies have suggested the antiviral potential of phytochemicals derived from *Artocarpus* species (32, 33). Among the four active crude extracts, DCM leaf extract of *Artocarpus heterophyllus* leaves, E16 emerged as the lead candidate due to its potent inhibitory effect and low cytotoxicity profile. Bioassay-guided fractionation of E16 yielded two highly active subfractions E16F4.3 and E16F4.4 with IC_50_ values of 0.43 μg/mL and 0.28 μg/mL, respectively. The other three extracts from leaves of *Artocarpus fratessi* (E13), *Artocarpus integer* (E22), and *Artocarpus sp*. (E40) also exhibited an antiviral activity against SARS-CoV-2, although they were not further investigated in this study. These findings demonstrate that leaves of the *Artocarpus* species plants have potential as a source of alternative or complementary medicine for the treatment of SARS-CoV-2 infection.

The mechanism by which the subfractions exert their antiviral activity remains unknown. We employed two assays to test the antiviral activity of subfractions, that is RT-qPCR assay and plaque reduction assay. E16F4.3 and E16F4.4 consistently showed potent activity in the two methods. Some subfractions—E16F4.2, E16F4.5, E16F4.6, and E16F4.7—also showed potent activity in the plaque reduction assay (Table III), despite showing little or no activity in the RT-qPCR assay at 1 μg/mL (Figure 1). In the plaque assay, virus was incubated for 1 hour with cells under presence of the subfractions, and then the plaques were counted after 4 days culture with subfractions. On the other hand, sustained activity is required to inhibit virus in the RT-qPCR assay in which virus remains for 6 days with subfractions. Because entry inhibitors are more likely to show higher apparent activity due to the removal of the virus after the wash step in the plaque assay, E16F4.2, E16F4.5, E16F4.6, and E16F4.7 might act during the infection stage prior to viral entry. However, identifying which stage is inhibited requires verification through experiments specifically designed to distinguish it, and this will be pursued following further separation of the subfractions.

In the extraction process, we employed successive sonication extraction, starting with hexane, a nonpolar solvent commonly used to extract nonpolar substances such as vegetable oils, fats, flavours, fragrances, colour additives, or other nonpolar bioactive substances (34–36). In our results, none of the hexane extracts from stembark or leaves exhibited antiviral activity against SARS-CoV-2, as indicated by RT-qPCR Ct value ranging from 7–13, which indicated no difference compared to the control (Ct around 12) (Table I). The second extraction solvent used was semi-polar solvent dichloromethane (DCM), which typically extract compounds such as phytosterols, terpenoids, quinones, phenolic substances (medium to low polarity), and alkaloids (37–40). Interestingly, our results demonstrated complete inhibition of SARS-CoV-2 infection (Ct >25 and CPE score 1, Table I) by four extracts (E13, E16, E22, and E40) obtained from leaves using DCM. Finally, the polar solvent methanol was used to extract a wide range of highly polar bioactive substances that could not be extracted with the previous solvents, including phenolic compounds (phenolic acids, flavonoids, tannins), alkaloids, terpenoids, saponins, glycosides, organic acids, and sugars (41, 42). However, none of the methanol extracts exhibited substantial antiviral activity against SARS-CoV-2 (Table I).

Compared with other plant-derived active extracts, the inhibition activity of E16 subfractions—E16F4.3 and E16F4.4—resulted in SI of >232.6 and >357.1 categorizing them as promising candidates relative to previous studies. For instance, andrographolide, the major component of *Andrographis paniculata* extract, exhibited antiviral activity against SARS-CoV-2 with an IC_50_ value of 0.036 μg/mL (0.034 μM), and CC_50_ value ranging from 13.2–81.5 μM in different cell types, resulting in SI >380 (43). Extract from *Artemisia annua* demonstrated antiviral activity against SARS-CoV-2, with median effective concentration (EC_50_) values of 83–390 μg/mL, varying by cell types (44). In the same report, the antimalarial drugs derived from the same plant, —Artesunate, Artemether, and Artemisinin—also showed antiviral activity against SARS-CoV-2 with EC_50_ values of 7–12 μg/mL, 53–98 μg/mL, and 151–208 μg/mL, respectively, and SI values ranging from 2–54 (44). Curcumin, a bioactive ingredient from turmeric root, has been reported to exhibit EC_50_ values of 7.9 μg/mL and 14.2–14.6 μg/mL, in CPE inhibition and RT-qPCR assays against SARS-CoV-2 (45). The traditional herbal medicinal product Echinaforce® (A.Vogel, Switzerland) derived from *Echinacea purpurea*, was reported to achieve complete inactivation of SARS-CoV-2 at 50 μg/mL (46). Glycyrrhizin from the root of licorice (*Glycyrrhiza glabra*), demonstrated antiviral activity against SARS-CoV-2 with an EC_50_ value of 0.44 mg/mL (47).

Even when compared with currently approved therapeutics for COVID-19 treatment, the antiviral activity of the subfractions from *Artocarpus heterophyllus* extract E16 remains comparable. For example, the broad-spectrum RdRp inhibitor Remdesivir has been reported to exhibit an EC_50_ value of 0.77 μM in a virus RNA replication assay, with SI of 129.87 (13). The 3CL protease inhibitor Nirmatrelvir (PF-07321332) demonstrated an EC_50_ value of 74.5 nM in a CPE inhibition assay (48). Similarly, the metabolite of the Molupiravir (EIDD-1931) showed IC_50_ values ranging from 0.45–2.5 μM against multiple SARS-CoV-2 variants, including Omicron derivatives, in a CPE inhibition assay (49). In our results, the subfractions F4.3 and F4.4 showed stronger inhibitory effect than 10 μM Remdesivir (6.0 μg/ml) even at the lower concentration of 1 μg/ml (Figure 1). However, dose dependent assays including cell cytotoxicity assay and plaque assays are required to substantiate their advantage. Detailed comparisons will be conducted in subsequent studies, in parallel with the further purification of the active compounds.

The future direction of this research is the isolation and identification of the active compound(s), as preliminary thin-layer chromatography profile indicated the presence of multiple substances in the subfractions which consist of chlorophyll, terpenoid, and flavonoid (data not shown). It is essential to isolate pure compounds with antiviral activity from these subfractions, although a synergistic effect of multiple compounds cannot be ruled out.

Further research is required to identify the compounds present in the two subfractions (E16F4.3 and E16F4.4) of *A. heterophyllus* leaf extract that were suggested in this study to exhibit antiviral activity against SARS-CoV-2, to elucidate the underlying mechanisms of action, and to evaluate the potential of these compounds as therapeutic agents for SARS-CoV-2. In conclusion, subfractions E16F4.3 and E16F4.4 from *A. heterophyllus* represent promising lead candidates for anti-SARS-CoV-2 therapy, exhibiting high selectivity indices (>232.6 and >357.1).

## Figures and Tables

**Table I:** Antiviral activity and cell viability of 54 extracts from *Artocarpus* genus plants

No	Code	Plant source		Solvent	Antiviral activity at 10 μg/mL			Percent cell viability at 100 μg/mL‡

					Real-time qPCR Ct value*		CPE score†	
		Species	Part		E gene	Orf^1^ab gene		
1	E1	*Artocarpus altilis*	Leaves	DCM	10.6 ± 0.3	10.9 ± 0.5	5	91.0 ± 1.6
2	E2			Methanol	9.2 ± 0.9	8.9 ± 1.5	4	125.4 ± 3.1
3	E3			Hexane	9.0 ± 0.7	8.0 ± 0.4	4	121.5 ± 2.9

4	E4	*Artocarpus elestica*	Stem/Stem bark	DCM	10.3 ± 1.6	9.3 ± 2.6	4	**30.2 ± 12.3**
5	E5			Methanol	10.0 ± 2.6	9.3 ± 2.9	5	116.8 ± 1.8
6	E6			Hexane	9.4 ± 0.3	8.2 ± 0.7	3	117.3 ± 4.4

7	E7		Leaves	DCM	11.3 ± 2.6	10.9 ± 2.6	3	**27.1 ± 4.4**
8	E8			Methanol	9.0 ± 1.4	8.2 ± 0.8	5	126.5 ± 2.7
9	E9			Hexane	ND	ND	3	117.9 ± 6.1

10	E10	*Artocarpus fretessi*	Stem/Stem bark	DCM	10.8 ± 3.9	9.7 ± 4.2	4	78.8 ± 6.2
11	E11			Methanol	9.2 ± 3.2	8.6 ± 4.2	5	116.0 ± 19.9
12	E12			Hexane	10.0 ± 1.7	8.9 ± 2.0	4	77.7 ± 2.7

13	E13		Leaves	DCM	**27.5 ± 0.2**	**27.0 ± 0.3**	**1**	108.8 ± 15.4
14	E14			Methanol	7.5 ± 0.7	6.9 ± 0.9	4	118.2 ± 21.8
15	E15			Hexane	10.3 ± 0.7	10.1 ± 0.4	4	110.8 ± 13.7

16	E16	*Artocarpus heterophyllus*	Leaves	DCM	**30.2 ± 0.9**	**29.6 ± 1.0**	**1**	130.9 ± 22.0
17	E17			Methanol	7.5 ± 0.1	6.8 ± 0.1	4	117.4 ± 16.1
18	E18			Hexane	10.4 ± 1.2	9.6 ± 1.0	4	109.5 ± 12.6

19	E19	*Artocarpus integer*	Stem/Stem bark	DCM	9.0 ± 1.9	8.4 ± 2.6	4	111.7 ± 6.0
20	E20			Methanol	7.7 ± 0.7	7.7 ± 1.5	5	91.5 ± 3.4
21	E21			Hexane	10.2 ± 0.8	9.5 ± 1.2	4	107.5 ± 3.2

22	E22		Leaves	DCM	**28.9 ± 0.2**	**28.6 ± 0.0**	**1**	99.2 ± 9.4
23	E23			Methanol	18.3 ± 15.8	17.8 ± 15.6	4	115.1 ± 1.9
24	E24			Hexane	8.9 ± 1.2	8.8 ± 1.5	3	90.0 ± 3.1

25	E25	*Artocarpus rigidus*	Stem/Stem bark	DCM	12.1 ± 0.4	12.1 ± 0.6	3	**21.4 ± 1.1**
26	E26			Methanol	8.9 ± 1.2	8.8 ± 1.4	4	97.8 ± 1.1
27	E27			Hexane	ND	6.8 ± 0.7	3	86.9 ± 0.2

28	E28		Leaves	DCM	ND	7.3 ± 0.8	3	**28.2 ± 10.7**
29	E29			Methanol	14.5 ± 10.0	14.4 ± 10.0	3	107.3 ± 4.0
30	E30			Hexane	9.3 ± 2.9	8.9 ± 3.6	4	91.3 ± 3.2

31	E31	*Artocarpus sericicarpus*	Stem/Stem bark	DCM	8.8 ± 0.4	8.6 ± 0.5	3	59.4 ± 2.1
32	E32			Methanol	7.2 ± 0.8	6.6 ± 1.1	5	100.6 ± 5.2
33	E33			Hexane	7.3 ± 0.5	6.7 ± 0.5	3	76.6 ± 1.3

34	E34		Leaves	DCM	8.2 ± 0.2	8.2 ± 0.3	4	104.8 ± 1.8
35	E35			Methanol	7.4 ± 1.2	6.8 ± 1.4	4	98.4 ± 1.8
36	E36			Hexane	8.9 ± 0.3	9.1 ± 0.4	4	90.0 ± 3.0

37	E37	*Artocarpus sp*	Stem/Stem bark	DCM	8.7 ± 0.9	8.4 ± 1.1	4	66.6 ± 0.7
38	E38			Methanol	8.6 ± 0.5	7.7 ± 0.3	3	82.4 ± 5.5
39	E39			Hexane	7.8 ± 0.0	7.3 ± 0.0	3	59.5 ± 1.7

40	E40		Leaves	DCM	**27.1 ± 0.6**	**27.0 ± 0.4**	**1**	51.9 ± 2.5
41	E41			Methanol	8.7 ± 0.3	9.0 ± 0.7	5	103.8 ± 4.3
42	E42			Hexane	10.9 ± 0.2	11.0 ± 0.3	4	83.5 ± 2.7

43	E43	*Artocarpus tamaran*	Stem/Stem bark	DCM	12.5 ± 0.8	12.5 ± 1.0	3	77.9 ± 2.0
44	E44			Methanol	12.2 ± 2.4	12.3 ± 2.3	5	91.5 ± 3.0
45	E45			Hexane	11.3 ± 0.8	10.7 ± 1.2	3	70.9 ± 1.6

46	E46		Leaves	DCM	8.3 ± 1.8	7.7 ± 1.8	5	**11.5 ± 0.6**
47	E47			Methanol	8.8 ± 0.7	9.6 ± 1.7	5	100.9 ± 1.7
48	E48			Hexane	12.6 ± 2.1	12.4 ± 2.0	5	91.6 ± 4.8

49	E49	*Artocarpus teysmanii*	Stem/Stem bark	DCM	13.0 ± 0.6	13.8 ± 0.6	3	73.8 ± 7.3
50	E50			Methanol	11.5 ± 3.6	12.0 ± 4.7	5	91.1 ± 1.8
51	E51			Hexane	13.5 ± 0.8	13.3 ± 0.8	3	90.9 ± 2.1

52	E52		Leaves	DCM	10.4 ± 0.2	10.2 ± 0.7	5	**10.6 ± 0.5**
53	E53			Methanol	8.2 ± 0.5	8.3 ± 0.6	5	88.3 ± 1.9
54	E54			Hexane	12.6 ± 1.0	12.5 ± 1.0	4	89.9 ± 3.7

0.01% DMSO					12.0 ± 2.8	11.6 ± 2.8	5	100

* Values represent the mean cycle threshold (Ct) ± standard deviation (SD) from two replicates. Values greater than 25 are shown in bold to indicate an active antiviral effect. ND: not determined due to experimental error.

† The Cytopathic effect (CPE) at 6 dpi was evaluated by observation and scored as follows: 1: 0–25% CPE; 2: 26–50% CPE; 3: 51–75% CPE; 4: 76–99% CPE; 5: 100% CPE. Score 1 is shown in bold to indicate an active antiviral effect.

‡ Cell viability was assessed using the MTT assay, and expressed as a percentage relative to 0.01% DMSO. Values less than 50% are shown in bold to indicate a cytotoxicity.

**Table II:** IC_50_, CC_50_, and Selectivity Index (SI) of subfractions of the E16 extract

Code	SARS-CoV-2		
	IC_50_ (μg/mL)*	CC_50_ (μg/mL)†	SI (CC_50_/IC_50_)
E16F4.1	93.37	>100	>1.1
E16F4.2	5.52	>50	>9.1
E16F4.3	**0.43**	**>100**	**>232.6**
E16F4.4	**0.28**	**>100**	**>357.1**
E16F4.5	2.96	>50	>16.9
E16F4.6	4.87	>100	>20.5
E16F4.7	5.04	>100	>19.8
E16F4.9	37.93	>100	>2.6

* The 50% inhibition concentration (IC_50_) was estimated from the RT-qPCR assay by plotting the percent expression level of the E gene 2^(−Ct)^ relative to the 0.1% DMSO control. The values represent the mean of two wells in a single experiment.

† The 50% cytotoxicity concentration (CC_50_) was estimated based on the analysis of Figure 2 as the highest concentration at which the cell viability exceeded 50%.

**Table III:** Inhibition percentage by subfractions in the plaque assay at 1 μg/mL

Code	Inhibition Percentage by plaque at 1 μg/mL*
E16F4.1	75.3 ± 3.9
E16F4.2	89.0 ± 1.0
E16F4.3	89.3 ± 2.4
E16F4.4	97.9 ± 1.0
E16F4.5	100.0 ± 0.0
E16F4.6	90.4 ± 2.9
E16F4.7	92.4 ± 1.9
E16F4.9	60.5 ± 2.4

* Data represent the mean inhibition percentage ± standard deviation (SD) from two replicate wells.

When the plaque count was zero, the inhibition percentage was recorded as 100%.
